# Supplementary material for: Are carbon emissions trading and green financial instruments synergistic? -Comprehensive quantitative research based on content analysis
Source: PLoS One. 2024 Mar 7;19(3):e0298601. doi: 10.1371/journal.pone.0298601 (PMC10919601; doi:10.1371/journal.pone.0298601)
Supplement: S1 Data — (ZIP) [file pone.0298601.s001.zip › Data/地级市Z_F工作报告.zip!/║╙▒▒/╨╧╠¿╩╨/└┤╫╘╣1⁄2╓┌║┼í╛┬φ┐╦╩2╛▌═°í┐_2015.docx]

各位代表：

　　现在，我代表邢台市人民政府向大会作工作报告，请予审议，并请各位政协委员和列席同志提出意见。

　　一、2014年的主要工作

　　刚刚过去的一年，是我市经济社会发展历史上极为困难的一年。在省委、省政府和中共邢台市委的正确领导下，我们始终坚持以经济建设为中心，以党的群众路线教育实践活动和“学讲话、转作风、优环境、促发展”大讨论活动为载体，统一思想、攻坚克难，稳增长、调结构、促改革、治污染、惠民生，经济发展稳中向好，社会事业全面进步。

　　——面对经济下行压力加大、企业生产经营困难、增速持续放缓的大形势，我们主动作为、苦练内功，经济运行总体保持平稳。初步核算，全市生产总值完成1668.1亿元、增长6%；全部财政收入174.4亿元、增长4.4%，增幅全省第四，公共财政预算收入95.7亿元、增长6.4%，增幅全省第六；社会消费品零售总额787.8亿元、增长12.4%。

　　——面对融资难融资贵、违法用地被国土部“约谈”、招商引资竞争剧烈的不利条件，我们统筹协调、攻坚克难，项目开工率和供地率分列全省第一、第三，固定资产投资1647.1亿元、增长16.2%；实际利用外资5亿美元、增长10%；新增规上工业企业185家，全市规上工业企业增加值完成610.2亿元、增长4.6%。

　　——面对偿债进入高峰、财政收入下降、收支平衡困难的巨大压力，我们未雨绸缪、挖潜增效，全年融资47.1亿元；民生支出236.1亿元、增长14.8%，占全部财政支出的80.2%，超过全省平均水平；城乡居民人均可支配收入分别达到19924元和8191元，增长9.5%和10%。

　　——面对环保标准不断抬高、污染治理成本激增和新《环保法》施行日益临近的艰难局面，我们多管齐下、强力减排，发挥政府和企业两个积极性，市区大气主要污染物PM2.5、PM10和二氧化硫浓度同比分别下降18.1%、20.1%、33.6%，二级及以上优良天数达到87天，比上年增加49天。

　　一年来，我们突出抓了以下工作：

　　（一）始终坚持以经济建设为中心，强化项目支撑，着力促进经济平稳较快发展。180项市重点在建项目完成投资350亿元；116个新开工项目全部开工，43个项目竣工或部分投产，8项省管重点项目完成投资69亿元。大项目、好项目增多，总投资10亿元以上的在建重点项目达到41个，占总数的23%；高新技术、先进装备制造及战略性新兴产业项目达到76个，占重点工业项目的53%。一是农业产能效益稳步提升。粮食再获丰收，总产444.8万吨，隆尧、宁晋获得“全国产粮大县”称号。智和、旺族等一批农产品加工项目开工或投产，新增家庭农场346家、合作社1000家、省级龙头企业16家，产业化经营率提高1.5个百分点。农业综合开发实现新突破，威县建成国家级现代农业示范区，新增省级示范区3家。二是工业转型升级步伐加快。传统产业的内涵和品质不断提升，全市工业投资完成1120.5亿元，增长14.4%，67个技改项目列入省专项投资计划，为全省最多。晶澳太阳能、北冶精密不锈钢等一批支撑项目投产。省级新型工业化示范基地达到8家、全省第一。六大特色产业集群完成工业增加值183亿元、增长5%，七大优势产业完成工业增加值410.1亿元、增长7.2%。汽车产业发展迅猛，红星汽车重组成功；中航长征汽车完成重卡商用样车研发；御捷车业取得工信部汽车整车生产资质和新能源汽车生产资质，全年销售电动车4.3万辆。三是现代服务业加速发展。新增小额贷款公司5家、挂牌上市公司15家，银行新增存贷比62.7%。居然之家、世贸天街等商贸综合体建成开业。新增省级电子商务示范基地2家、中国“淘宝村”13个，数量均居全省第一，2个电子商务平台上线运行。成立市软件与信息服务业协会，北方交通信息产业园开工建设，举办了首届软件产品展览会，18家企业参会，备案软件产品达65个。全国玻璃期货基准价区调整到沙河，我市在玻璃行业的地位得到质的提升。46个旅游重点项目完成投资19.7亿元，全市旅游业总收入突破100亿元。服务业完成增加值558.2亿元、增长8.1%。

　　（二）始终坚持以改革开放为抓手，强化创新驱动，着力提升发展的活力和水平。一是各项改革深入推进。印发了市政府13方面55项职能改革任务，对市直52个涉批部门保留的1035项权力在《邢台日报》公布。下放和减少市本级行政职权576项，向两区和市开发区分别下放42项、31项，公布行政审批200项，建立了统一规范的网上审批、并联审批，流程优化率达到82.6%，对47个市直部门实时动态监控。威县被省政府确定为县级综合改革试点，集中26个部门166项职权的审批局开始运行。邢家湾、莲子镇、西郭城被列为全省经济发达镇行政管理体制改革试点，滏阳经济开发区升级扩规获批，开创了全省跨区域整合的先河。平乡土地承包经营权确权颁证试点工作整县推进顺利。二是开放招商步伐加快。商务、海关、检验检疫等紧密合作，邢台外向型经济格局初步形成，外贸出口逆势而上，总值达15.6亿美元，增长29%。首创“工商顾问制”，推行课题式监管，市场活力进一步增强，新增市场主体6.5万个；其中外资企业36家、增长140%。全年签约重点招商项目126个，其中投资超10亿元的39个。积极对接京津，围绕医疗、教育、科研等实施“1+1”招商，与15位院士、17家院校、33家医院达成合作，既改善了公共服务软件，又搭建了招商引资平台。三是科技创新实现突破。市财政安排1000万元专项支持科技型中小企业，省级科技型中小企业达到1100家，市科技企业孵化器建成，新增院士工作站2家、省级以上科技创新平台12家。继续实施“质量提升三年行动计划”，新增省名牌产品48项、省优质产品52项、中国驰名商标3件。

　　（三）始终坚持以大气治理为重点，强化标本兼治，着力改善生态环境和城市形象。一是抓企业达标治理。以9月底为完成时限，倒排进度，逐厂施治，投入37.6亿元，实施治理项目2860个，深度治理企业1815家，钢铁、化工、玻璃等重点行业均实现达标排放。二是抓面源污染整治。制定了扬尘治理18条标准，全面实施“挡、浇、洒、盖、清、硬、洁”措施，建筑扬尘大幅下降。淘汰10蒸吨及以下燃煤锅炉535台，建设洁净型煤配送中心17个，销售型煤近10万吨，灭失和拆解“黄标车”2.3万辆，637家加油站完成油气回收治理，35家工业企业完成挥发性有机物治理。三是严格监管执法。在市区安置了11块监控屏幕，对106家重点企业统一在线监测，实时接受社会监督；实行网格化管理，全年查处涉嫌环境违法犯罪案件159起、违法企业651家。我市治理大气污染的做法得到全国人大执法检查组和国务院、环保部的肯定。

　　（四）始终坚持以规划设计为引领，强化城乡统筹，着力提高城市品位和解决遗留问题。一是围绕做大做强中心城市，聘请清华、同济大学编制和提升“一城五星”、“四带一片”等系列规划；扎实推进“四区”建设，龙岗片区按PPP模式启动开发，七里河片区引进了碧桂园开发项目。以国家土地例行督察为契机，深入推进“双违”整治，整改违法占地3.5万亩，收回土地1636亩，困扰多年的违法占地乱象得到彻底治理，受到国土部好评。实地踏察、现场办公、全面梳理城建遗留问题，规范“四改”项目建设，整合项目32个，开工回迁房项目26个。加快市政基础设施和公共服务设施建设，市博物馆开工，打通新华路北延、兴达路南延等5条断头路，建设大街地道桥提前半年通车，完成11条小街巷整治和103个老旧小区物业管理提升。二是围绕县城和乡村建设，完成16个县（市）城乡总体规划编制，579项县城建设重点工程完成投资130.8亿元，新河建成省级园林县城，南和通过国家园林县城初评。启动农村面貌改造提升省级中心村示范点14个，示范点和争取省级财政奖补资金均为全省第一；169个省级重点村完成投资10.1亿元，改水、改厕等15件实事全部完成。三是围绕完善基础设施，邢衡高速、京港澳高速改扩建工程建成通车，环城高速实现联通。邢衡、邢临两条高速经营权及债务转让工作取得重大进展。邢和铁路注册地确定在我市。热电联产项目取得国家能源局“路条”，初步完成场址勘测。南水北调主体工程正式通水，17个县（市）配套水厂全面开工。集中提升园区建设水平，22家省级园区实现“十通一平”，14家市级园区实现“七通一平”。

　　（五）始终坚持以民生改善为目标，强化社会公平，着力推动经济社会协调发展。一是努力为群众办实事。争取国开行棚户区改造贷款51.4亿元，开工建设保障性住房和棚户区改造住房1.88万套、竣工1.36万套，改造农村危房8200多户。城镇基本医疗保险实现市级统筹，企业职工养老保险提高199元/月，城乡低保标准分别提高42.5元/月和163元/年，乡级人力资源社会保障公共服务平台实现全覆盖，社区工作人员工资提高40%以上。新增城镇就业6.55万人，转移农村劳动力6.4万人（次）。多渠道解决供暖问题，投资5000万元改造邢东热电厂，铺设和改造管网12.6公里，采用远程监控、无线采温、数字调峰技术，供暖能力和质量稳步提升。市儿童福利院主体完工。新增社区居家养老服务中心44所、农村互助幸福院553所、计生养老家园60家，我市被列为全国计生养老试点。设立100万元大学生励志成才专项助学金，筹集福利彩票资金175.7万元，资助1315名贫困家庭大学生。深入开展“春雨行动”和“走千连万”活动，3.5万户贫困家庭有了持续增收项目，15.8万人稳定脱贫，内丘脱贫出列。解决了29.5万农村人口饮水安全问题。二是全面发展社会事业。依法规范市区住宅区配套幼儿园中小学建设，新建改扩建幼儿园、中小学100所；投资5400万元的主城区基础教育信息化城域网完成招标；邢台医专老年养护中心主体完工，皇寺校区部分投用。市人民医院新院区项目奠基，中医院迁建、三院改扩建等项目顺利推进，眼科医院升格为河北省眼科医院并迁入新址。设立100万元政府文艺精品创作奖，支持了河北梆子《吕玉兰》等一批文艺作品。新增省级文化产业示范园区4家、国家级非物质文化遗产项目3项，完成了大运河（邢台段）世界文化遗产保护申报工作。成功举办第四十三届世界技能大赛全国选拔赛和省第八届残运会暨第四届特奥会，国家级高水平体育后备人才基地通过验收。三是倾力建设平安邢台。开展了食品安全三大攻坚战，推广了校长陪餐制、食品安全责任险等有效措施，食安工作走在全省前列。坚持安全生产隐患一周一曝光，开展审计解剖式执法检查、警示约谈、标杆创建等系列活动，安全生产监管水平进一步提升。妥善处置道达尔油品事件，维护了6500多名车主的合法权益。原中达集团案件取得重大进展，国有资产流失得到挽回。公安刑事办案“三统一”机制全国推广，在全省率先实施“一村一法律顾问”制度，信访稳定、社会治安综合治理常抓不懈，社会大局和谐稳定。双拥共建深入开展，征兵工作实现了登记率、合格率、直招率三个全省第一，首次无一例责任退兵。造林绿化、水生态保护、民族宗教、外事侨务、老龄妇幼、防震减灾、应急管理、统计审计、邮政通信、地方志、档案等工作取得了新业绩。

　　（六）始终坚持以自身建设为根本，强化素质提升，着力提高政府公信力和执行力。一是抓学习、提素质。继续坚持市政府常务会议学习制度；专题学习列入部门办公会议日程，科长以上干部全部建立工作日志；“学、转、优、促”大讨论活动深入开展，对发展平台、新兴产业等课题开展了深入研究。二是抓公开、促规范。在全省率先搭建“四大平台”，对22322名执法人员实行了动态监管，清理不具备执法资格人员3797人，行政执法依据、内容、流程、责任全部公开。实行公民列席政府常务会议制度和政府参事制度，发布政府信息4万余条。畅通群众诉求表达渠道，受理群众来电、来信、行政复议申请6.8万件。办理代表建议、政协提案1252件，按时办结率100%。三是抓整改、转作风。深入开展党的群众路线教育实践活动，开门整改“四风”，公布“问题清单”，明确标准和时限，逐条加以改正，仅市政府督查室现场督查427次、2366人（次），762项工作事事有结果。全面清理和处置违规公务用车、超标办公用房，有效解决租房办公问题，“三公”经费压减6300万元，下降16.4%。

　　在较好完成年度任务的同时，我们坚持把各项工作往深里做、往实里做、往细里做、往群众身边做，破解了一批阻碍发展的瓶颈，取得了一些理旧账、打基础、管长远的制度成果。各位代表、同志们，回望渐渐远去的2014年，以下五条经验弥足珍贵：一是必须坚定信心。艰难时期，信心比黄金还重要。2014年可以说是邢台历史上最困难的年份，全体干部、全市人民的坚定信心是我们战胜困难的第一法宝。二是必须保持定力。全面建成小康邢台的任务书、路线图、时间表已经确定，任何艰难风险、任何干扰和挫折都不能使我们偏离方向、降低标准、停止脚步。任他东南西北风，咬定发展不放松。三是必须领导带头。干部就是一切，领导至关重要。上级党委、政府和中共邢台市委的正确领导是根本保证，各级、各部门、各单位领导同志始终走在发展的最前列，干在工作的第一线。四是必须依靠群众。群众是真正的英雄，人民是永远的靠山，只要心系群众、团结群众、服务群众，就能凝聚起振兴邢台的强大合力。五是必须锲而不舍。天上不会掉馅饼，小康社会等不来。每一项成绩的取得都来自几十次、上百次的督导，每一次的胜利都出现在再一次的坚持之后。可以说，2014年邢台的干部群众是蛮拼的。在这里，我代表邢台市人民政府，向全市人民，向人大代表、政协委员，向各民主党派、工商联、无党派人士和人民团体，向驻邢人民解放军、武警官兵、政法干警和驻邢各单位，向关心支持邢台发展的海内外朋友，致以衷心的感谢和崇高的敬意！

　　毋庸讳言，我市总量小、增速慢、结构差、效益低、不平衡、包袱重的局面没有根本改变，一些矛盾又碰头叠加，相互交织，日益严重。一是财政收支矛盾。一方面，财政收入增速下降，减税政策频出，过剩产能压减，企业成本居高，可用财力严重不足；另一方面，还债处于高峰期、社保水平逐年提升、民生支出扩面提标、养老改革和配套资金等导致刚性支出快速增长。二是既定的任务目标和连年增速下降矛盾。由于经济下行、结构调整、治理污染等原因，主要经济指标增速连续两年未能达到全省平均水平，这意味着我们同全国、全省差距仍在拉大，今后任务更加艰巨，倒逼压力有增无减。三是新旧动力转换矛盾。邢台传统产业比重大、层次低，发展的动力逐渐衰减，而劳动力素质低、科技投入低、发展路径依赖又导致创新艰难、动力不足，动力不足、转换缓慢和青黄不接是发展缓慢的主要原因。四是公务员队伍建设同时代发展要求的矛盾。公务员素质、能力和作风同党的要求和群众期盼差距较大，法治观念淡薄，懒政、怠政、不作为现象仍然存在，廉政建设任重道远。特别要向大会报告的是，由于各种原因，年初确定的部分指标未能如期完成，28项重点工作未达到进度要求。对此，我们一定认真分析，强化督导，尽快完成。

　　二、2015年的主要目标和工作重点

　　今年是全面深化改革的关键之年，是全面推进法治邢台建设的开局之年，也是“十二五”规划的收官之年，全市经济社会发展的总体要求是：全面贯彻中央和省一系列方针政策，按照市委八届六次全会的决策部署，主动适应经济发展新常态，坚持“稳中求进、好中求快”主基调，以提高经济发展质量效益为中心，围绕“三个发展”，主攻“四个战略重点”，深化改革开放，突出创新驱动，加强民生保障，推进依法治市，努力保持经济又好又快发展和社会和谐稳定。

　　主要预期目标：全市生产总值增长8%；全部财政收入增长7.5%，公共财政预算收入增长9%；固定资产投资增长18%；规模以上工业企业增加值增长7%；服务业增加值增长10%以上，社会消费品零售总额增长12%；出口总值增长5%。单位生产总值能耗下降3%，化学需氧量、二氧化硫、氨氮、氮氧化物排放量分别削减0.1%、0.4%、1.6%、5.58%，PM2.5浓度下降5%。城乡居民人均可支配收入分别增长8%和8.5%；城镇登记失业率控制在4.5%以内；人口自然增长率控制在8.8‰以内。

　　上述目标是在优化经济结构、压减过剩产能、提高环保标准、坚持以质量效益为中心的前提下，科学审慎确定的，总体高于全省平均，但也仅仅是工作的底线。因为我们无论地理区位、资源禀赋，还是产业基础、基础设施，都是全省中游，没理由发展慢；同全省、全国的发展差距仍在不断拉大，要如期实现全面建成小康社会的既定目标，不允许发展慢；经过多年持续努力，主要瓶颈均已打破，前进的基础更加牢固，完全具备把差距转为成长空间的可能，实现超常规发展的条件成熟。能否乘势而上，就看我们是否努力了。我们一定要拼出真力、形成合力、中场发力、持续用力、竭尽全力实现赶超，特别要奋发有为地做好今年各项工作。

　　（一）进一步深化改革、释放红利，激发经济社会发展活力。一是抓好重点领域改革。继续落实好2014年中央出台的改革任务，做好2015年55项改革的衔接准备。市级改革全面推进，完成市、县政府机构改革和机关公车制度改革任务，推进机关事业单位养老保险制度改革。推进公共资源交易规范化，做到交易活动“八统一”。实施国企改革攻坚年，做好中达的善后工作，撤销15家破产企业清算组。全力支持威县综合改革探索，推广行政审批、园区管理、城乡一体化、农村产权等专项改革做法，鼓励各县各部门“近水楼台先得月”，防止“墙里开花墙外香”。强力推进邢家湾、莲子镇、西郭城三镇融合发展，探索三镇行政管理改革新思路。深化农村综合改革，抓好21个重点村集体产权股份合作制改革，新建5个县级农村产权交易平台，全面完成平乡土地承包经营权确权登记颁证任务。发挥好市政府投融资管理中心作用，实现顺德集团战略转型。二是进一步简政放权。对国务院去年下放的708项权力进行“回头看”，确保放下去、用得好、不反弹。衔接好今年国家、省下放的行政审批事项，继续推进市向县、县向乡放权。完善网上联合审批，提高审批效率。落实市场准入负面清单制度，全面放开竞争性经营行业和投资领域，全面清理非行政许可审批和前置审批。继续编制公布政府部门权力清单、监管清单。三是掀起全民创业新高潮。认真落实支持民营经济发展10条措施，开辟登记注册、审批核准、环评审批等绿色通道；财政列支100万元，支持企业管理人员培训；实施“三个三十”工程，充实中小企业信用担保资本金，建设1家省级创业辅导基地，启动大学生创业园区建设。学习沙河“金融生态”创建经验，服务企业和银行对接。顺应政府投资方式变化，争取中央和省政府引导性创投基金、产业引导基金支持；支持26家上市公司通过增发、配股、股权质押等途径，继续在资本市场再融资。

　　（二）进一步抓住项目、加大投入，培育新的经济增长点。一是加大项目建设推进力度。安排市重点在建项目200个，年度投资370亿元以上，列入省重点项目数量不低于全省平均水平；产业项目投资力争增长25%以上。继续坚持领导分包和月通报、双月调度等制度，强化项目核心投资考核，学习隆尧“项目投产问效年”经验，抓项目投产企业入统，抓群众就业，抓财政增收。确保87个续建、113个新开工项目按时完成进度，中航特种合金材料、先腾光刻机、明盾高性能材料、万达轮胎、地井塑业等50个项目竣工或部分投产。河北迈普润光学晶体、卓达新型建筑材料、谢雷酒品包装产业园、光明食用菌等重大前期项目力争提前开工。加强项目谋划，每个县（市、区）至少谋划1个50亿元以上项目，全市项目储备保持在500项以上。二是提高项目招商实效。学习邢台县县级干部人人肩上有引资任务、平乡“项目上、干部上，没有项目岗位让”做法，组织100个精干团组定点招商，邀请100家企业来邢对接，签约100个重点项目；立足现有产业，优化软环境、动员全社会、依靠企业家、围绕产业链，继续开展“1+1”招商、“一二三产”混业发展，实际利用外资5亿美元。加快对接京津步伐，继续在教育、医疗、科研等领域同其它市错位竞争，力争再达成一批合作。探索推行PPP等合作模式，创新财政投入方式，撬动和引导社会资本加大投入。三是加强项目质量管理。学习市开发区“八项必考察、七项评估”经验，科学评估项目质量，着力提高技术水平、产业关联度。改进建设用地供应机制，优先支持大项目、好项目，逐月跟踪、动态调整，激励高效使用土地要素。学习临西“以重点企业为核心，引进上下游关联企业和核心技术，带动产业集群发展”经验，进一步明晰各县、各园区主辅业，深入实施“飞地”政策，引导项目聚集，延长产业链，形成新集群。

　　（三）进一步转型升级、创新驱动，优化产业结构。一是加快传统产业改造升级。始终把七大产业、六大集群作为产业发展的重中之重，制定主导产业发展规划。支持邢钢短流程改造，支持龙海、吉泰重组，继续支持旭阳工业园对外合作、上档升级。抓好“三百”工程，推进“两化”融合，加快好孩子、超威、中玻、丁腈等续建项目。实行月调度、季分析，加强对工业经济运行的监测与调度，力争新增规上工业企业150家以上。二是加快培育新兴产业。学习清河“高端化引领、园区化承载、城市化带动”经验，力促产业向中高端迈进。安排1000万元专项资金，支持新能源汽车产业发展；红星汽车恢复生产，完成1万辆整车产销；德动汽车一期正式投产，形成5万辆的生产能力；御捷车业产销10万辆。中航长征中重卡整车项目竣工投产，北汽轻卡合作项目加快推进。抓好全国新能源示范城市创建工作，加快宁晋、市开发区光伏产业基地建设，支持晶龙等龙头企业延长产业链、提升带动力。继续支持光热企业发展，大力推广新能源产品的应用。三是加快发展现代服务业。聘请专业人员担任主管金融的副县（市、区）长，总结桥西区金融招商经验，推动邢台银行走出去，扩大规模，域外金融机构引进来，至少增加1家分行，力争1家农信社改为农村商业银行，争取村镇银行发展实现新突破，引导小额贷款、担保公司等规范发展，新增挂牌上市公司20家以上。完善提升现有3家省级物流聚集区，力促绿旗国际物流港、平乡内陆港开工建设。做好电子商务进农村清河试点工作，建设10个县域特色产业电子商务平台。整合旅游资源，大力发展山水风光游、休闲农业游、历史文化游，年内推出古村落游等特色线路，争取实现旅游业总收入130亿元。引导社会资金，启动推进东方太极缘文化产业园等项目。积极发展服务外包、健康养老、快递服务等新兴服务业。四是强化科技支撑。学习临城引入彭苏萍院士经验，加强产学研合作，力争每县建立1家院士工作站或特色产业技术研究院；实施“222”人才引进工程，带动专业团队、科研成果和高科技项目引进；鼓励科技人员和社会资本创办科技型企业，市财政继续列支1000万元，以引导基金方式专项支持科技型中小企业；光源太阳能中温集热管等6个项目争取列入省科技成果转化项目；力争新增省级科技型中小企业500家、高新技术企业10家，规上企业高新技术产业增加值增长15%。完成“质量提升三年行动计划”，争创省名牌产品40项。

　　（四）进一步夯实基础、提质增效，推动“三农”快速发展。一是着力优化农业结构。突出抓好粮食生产核心区建设，粮食总产稳定在450万吨左右。加快畜牧、蔬菜、林果三大产业发展，新建市级畜禽养殖标准化示范场20个，新发展蔬菜面积3万亩，果品总产达到140万吨。巩固特色高效农业示范园区建设成果，不断完善提升，扩大辐射带动能力。高起点规划、高标准推进生态经济沟建设，集中打造10个示范区。二是推进农业产业化经营。加大农业对外开放力度，学习柏乡食品加工业专题招商做法，每个县（市）高标准建设1-2个现代农业园区。加大产业龙头引培力度，每个县（市）至少引进1个投资超亿元产业化项目，突出抓好智和、旺族、谷养谷、婴泊等项目，力争新增市级龙头企业30家以上、年销售收入5000万元以上8家，农业产业化经营率提高1.5个百分点。三是改善农村生产生活条件。抓好南水北调配套工程，水厂和管网设施6月底前建成投用；结合地下水超采治理和引黄配套工程建设，启动全市生态水网建设规划编制工作，发挥河道、水库等水利工程综合功能；加快滞洪区安全建设；发展节水灌溉面积25万亩，建设高标准农田5万亩。改善农村公共设施，继续实施农村面貌改造提升，完成129个省级重点村和10个中心村示范点建设，新改建农村公路400公里。四是加快农民脱贫致富。推广内丘“县有龙头、村有组织、户有项目”经验，大力实施精准扶贫、产业扶贫，学习广宗“引进教授工作站”做法，大力实施人才扶贫、智力扶贫，加快799个重点村整村推进工作，为5万户农村贫困家庭建设持续增收项目，实现20万人稳定脱贫。

　　（五）进一步依靠法律、强力治污，促进环境持续好转。广泛发动群众，全面执行新《环保法》，对环境违法行为始终保持高压态势，形成科学治污、全面治污的强大合力。一是巩固大气污染防治成效。把“一城五星”的治理措施扩展到全市，继续实行领导分包污染企业，实施煤电节能减排改造升级，推广“近零排放”、微煤雾化等技术，开展企业清洁生产试点工作；加强在线监测，保障治污设施高效运行。深入治理面源污染，广大农村推广南宫联村秸秆气化站供暖做法，“一城五星”继续按“一户不落、一炉不差”目标，加大洁净型煤推广力度，淘汰全部黄标车，有效控制扬尘污染，深度治理挥发性有机物。旭阳余热供暖和SNG项目建成投用，动工建设邢台热电联产、桑德循环经济产业园、邢台新蓝天氢能源汽车等项目，年内力争完成50%以上的工作量。二是实施水环境综合治理。对16条劣V类水质河流进行全面治理，逐河排查污染源，制定治理方案，在《邢台日报》公布河段分包领导名单，严格执行断面考核制度，力争一年见效，两年达标，三年水网还清。扎实推进全国水生态文明城市试点工作，加强饮用水源地保护，深度治理地下水超采，压采2.36亿立方米。三是提高造林绿化水平。尊重实际、科学植树，以浅山丘陵区及河流沿岸为重点，完成造林面积26万亩，森林覆盖率提高1个百分点。学习南和苗圃式绿化经验，大力发展林果业，继续把核桃、苹果作为重点，力争苹果销售15亿元。加强管护，严防森林火灾，巩固生态建设成果。

　　（六）进一步完善功能、提升品质，提高城镇的承载力和美誉度。一是建设“繁荣、舒适”中心城市。统筹推进“一城五星”建设，启动联通道路改造提升，完成绿化、亮化、畅化、洁化、美化。主城区启动“品质提升三年行动计划”。“四区”开发，完善规划、加快建设；“五带”改造，统筹京广铁路及环城4条主干道，在招商合作上争取有大的突破。学习桥东区“一年两次百日大会战”做法，谋划建设市区新环城公路，实施中兴路电闸箱整顿迁建工程。抓好一批重点工程，推进滨江路、新华路、团结大街、建设大街改造，加快市博物馆、生活垃圾备用填埋场建设，实施白马河综合整治，加快顺德路东片区、育英街片区等棚户区改造，妥善解决居民安置问题。启动邢台市智慧城市建设。二是提升城镇建设管理水平。学习宁晋等地违法占地整治经验，全面贯彻《国土法》，彻底完成国家土地例行督察整改任务。对各县（市、区）土地指标使用情况进行清查，提高土地要素使用效率。注重内涵式发展，强化“里子”意识，向管理要效益、要水平。出台《邢台市全面提升县城建设层次和水平工作方案》，启动县城“扩容升级三年行动计划”。加强县城交通、城管、交管、天网工程等整合共享，完善网络基础和综合信息平台。推广任县城乡垃圾一体化处置经验，南和通过国家园林县城验收。加强新增补24个镇的建设管理，开工滏阳“三镇”医院、道路、科技企业孵化器等15个项目。三是加快推进基础设施建设。争取京九客专南延到邢台境内；搬迁长途客运站，建设高铁综合枢纽；力争邢台机场改造、邢和铁路项目开工，抓好邢石大道、308国道北段、邢左线、京港澳高速连接线等12条线路建设改造，加快邢汾高速大峡谷旅游连接线等项目前期，谋划轨道交通等大容量公共交通项目。保障特高压电网项目顺利建设。提高城市防洪标准，统筹利用长江水、黄河水、水库水和中水，做好邢台水文章。省级园区达到“十一通一平”，市级园区达到“八通一平”。

　　（七）进一步改善民生、统筹兼顾，促进社会事业协调发展。一是着力保障群众的基本生活条件。千方百计稳定和扩大就业，力争新增城镇就业5.7万人，下岗失业人员再就业1.5万人，转移农村劳动力5.9万人（次）。继续推进城乡低保扩面提标，城乡居民养老保险参保率稳定在90%以上。推广巨鹿“医养一体、两院融合”模式。扩大贫困家庭大学生资助面。市救助站完成扩建，市儿童福利院投入使用。扎实推进保障房建设，开工1.93万套、竣工1.5万套，完成省下达农村危房改造任务。解决41.8万农村人口饮水安全问题。铺设供暖管网复线5.1公里，进一步提高供热水平。“三级平台”实现全覆盖，彻底解决个别农村、社区无活动场所或不能正常服务群众问题。二是大力发展各项社会事业。改造建设学校150所，新增标准化中小学100所，加快建设技师学院现代服务业产教园区，创建2个省级中等职业教育实训基地。邢台医专晋升河北护理学院工作实质启动，皇寺校区老年养护中心投用。加快市人民医院新院区、中医院迁建、三院改扩建等项目建设，完善提升县级医院和乡镇、社区卫生院，提高医疗卫生服务能力。辰光装饰城完成设施验收移交和恢复体育功能的规划设计；加快解决运动员入学难、安置难等实际问题；举办市第十四届运动会。进一步加强文物保护，全面铺开《邢台市志》编纂工作，搞好修志编鉴和方志资源挖掘利用。三是全力维护社会稳定。学习新河“邀请省市专家参与季排查制度”做法，抓好安全生产，遏制重特大事故发生。深入开展食品药品隐患排查和专项整治，继续加强学校食品供应专项治理。强化金融监管，加强诚信建设，建立金融风险预警和执法协作机制，严厉打击非法集资。推广大曹庄“一村一图一户一情”平台建设经验，加强基层网格化管理，依法加强信访工作，严厉打击各类违法犯罪行为，促进社会和谐稳定。加强国防教育和国防后备力量建设，大力开展双拥共建活动，切实提高军转安置和优抚工作水平。民族宗教、外事侨务、老龄妇幼、人口计生、防震减灾、统计审计、应急管理、邮政通信、档案等工作取得新进步。高质量编制“十三五”规划，为长远发展奠定基础。

　　各位代表，让广大人民群众共享改革发展成果，不断提高生活水平，是政府义不容辞的责任。今年将精心办好一批民生实事：⑴治理“四河”。按照“十化”标准，启动市区牛尾河、小黄河、茶棚沟、围寨河综合治理，力争一年还清、两年建成、三年完善。⑵整治市区小街巷。改造西门里街中段、拥军街、石油巷等17条街道路灯，对永祥街、新东街等10条街道进行治理。⑶改善公共厕所条件。统筹规划市区公共厕所，新建改建100座，提升保洁服务水平。⑷共建优质中小学。引进名校教育资源，合作建设北京师范大学邢台附属学校、北京第二外国语学院邢台外国语学校。⑸建设基础教育城域网。主城区建成投用，“一城五星”和宁晋、柏乡实现联网。⑹提升养老水平。建设农村互助幸福院553个、社区居家养老服务中心13个、计生养老家园20个，五保集中供养能力达到70%以上。⑺拓展物业服务。在去年基础上，再拓展100个居民小区，提升物业服务水平。⑻开展“交通秩序整治年”。优化交通组织，完善交通设施，开展交通违法行为专项整治，市区达到交通秩序管理二等水平。⑼完善体育健身设施。开工建设市水上运动中心和2个省级体育公园，建设和完善县级全民健身场馆10个，在20个乡镇、100个社区、600个村修建健身场地，在40个公园、广场、游园开辟健身区。⑽文化惠民。举办百场文化惠民演出、百支群众舞蹈队大展示、百名文艺工作者下基层活动。⑾延伸金融服务。POS机等银行服务终端实现村村全覆盖，方便村民办理小额取现、转账等业务。⑿农村清洁能源利用。推广1600户农村沼气、4.5万户清洁燃烧炉具以及多种清洁能源替代模式。⒀建设“农民工之家”。组织建筑领域开发单位、建设单位和施工队伍100家以上，开展技能培训、法律咨询等服务，维护农民工合法权益。

　　三 、 加强政府自身建设

　　各位代表、同志们，综合分析内外形势，2015年邢台比2014年面临的困难会更多、任务更艰巨、情况更复杂，而能否做好今年的工作，又攸关“十二五”规划的完成，攸关本届政府既定目标的实现，攸关邢台全面小康社会的建设。必须加强政府自身建设，在以下三方面起带头作用。

　　（一）带头全面深化改革。邢台是在改革开放中快速发展起来的，也是在改革开放的推进中同其它地区拉开距离的。在当前深化改革新的历史机遇面前，政府的态度至关重要，决定着当前和未来的发展速度和质量。一要不断加深对改革的认识。时刻牢记改革是新的伟大革命，是当代最鲜明特色，是决定命运的关键抉择，是赶上时代的重要法宝。二要做改革的最坚定拥护者、执行者。改革的核心问题是处理好政府和市场、社会的关系，就是解决政府越位、缺位、错位问题，就是政府被改革。面对利益调整，要自觉做到个人利益服从集体利益、部门利益服从全局利益、眼前利益服从长远利益，不喊空口号，不作旁观者，不搞中梗阻，不能“大撒手”。三要让改革红利尽快惠及邢台群众。改革怎么样，群众说了算。对上级的改革措施要即知即行，防止拖延迟滞；要不折不扣，防止缺斤短两；要上下贯通，防止衔接不畅；要加强事中事后监管，防止一放了之、放了接不住、重放轻管等。总之，要让人民群众和市场主体受到尊重，感到便利，降低成本，看到进步。

　　（二）带头全面推进依法治市。市委八届六次全会确定全面推进法治邢台建设，这是落实中央十八届四中全会和省委八届九次全会决定的重大举措。法治政府建设是依法治市的关键环节，必须走在前面。一要坚持依法决策。增强法制观念，养成法治思维，坚持重大决策公众参与、专家论证、风险评估、合法性审查、集体讨论决定，健全政府参事、法律顾问制度，建立决策事项备案和决策责任终身追究制度，提高决策的法治化水平。二要坚持依法行政。明确权力清单、监管清单、负面清单，做到法定职责必须为、法无授权不可为，坚决纠正不作为、乱作为，坚决克服懒政、怠政，坚决惩处失职、渎职，做到制度科学、程序正当、过程公开、责任明确。三要自觉接受监督。以公开为常态，以不公开为例外，坚决推进决策公开、执行公开、管理公开、服务公开、结果公开，主动接受人大、政协、舆论和社会监督，消除权力寻租的空间，让政府权力始终在阳光下运行。

　　（三）带头全面加强党的建设。党的领导是中国特色社会主义最本质的特征，是做好政府各项工作的根本保证。一是市政府党组要严格按照党章要求，在市委的集中统一领导下，围绕中心，谋划大局，突出重点，狠抓落实。要强化党组领导核心作用，适应党建新常态。要严格党内政治生活，加强党风廉政建设，干净干事干成事，营造风清气正的政治生态。二是政府系统广大党员干部，要带头遵守党的政治纪律和政治规矩，在思想上、政治上、行动上同党中央、省委、市委保持高度一致，在克难攻坚、推进邢台发展的实践中发挥模范带头作用。三是各级政府及部门要进一步巩固和拓展“为民、务实、清廉”教育实践活动和大讨论活动成果，把思想建设、组织建设和作风建设贯穿落实全过程。

　　各位代表！时代的号角催人奋进，邢台的崛起任重道远。让我们在省委、省政府和中共邢台市委的坚强领导下，以坚韧不拔的信念、只争朝夕的精神，万众一心、开拓进取，为建设全面小康的邢台、富裕殷实的邢台、山清水秀的邢台而努力奋斗!
